# Membrane free-energy landscapes derived from atomistic dynamics explain nonuniversal cholesterol-induced stiffening

**DOI:** 10.1101/2023.02.02.525347

**Published:** 2023-02-03

**Authors:** Giacomo Fiorin, Lucy R. Forrest, Josè D. Faraldo-Gómez

**Affiliations:** a National Institute for Neurological Disorders and Stroke, Bethesda, MD, USA; b National Heart, Lung and Blood Institute, Bethesda, MD, USA

## Abstract

All lipid membranes have inherent morphological preferences and resist deformation. Yet adaptations in membrane shape can and do occur at multiple length scales. While this plasticity is crucial for cellular physiology, the factors controlling the morphological energetics of lipid bilayers and the dominant mechanisms of membrane remodeling remain unclear. An ongoing debate regarding the universality of the stiffening effect of cholesterol underscores the challenges facing this field particularly for lipid mixtures, both experimentally and theoretically. On the computational side, we have argued that enhanced-sampling molecular dynamics simulations are uniquely suited for quantification of membrane conformational energetics, not only because they minimize a-priori assumptions, but also because they permit analysis of bilayers in deformed states. To showcase this approach, we examine reported inconsistencies between alternative experimental measurements of bending moduli for cholesterol-enriched membranes. Specifically, we compute bending free-energy landscapes for multiple bilayers of sizes up to ~2,000 lipids, using steady-state all-atom simulations of both unperturbed and deformed morphologies, totaling over 100 microseconds of sampling. This enhanced simulation approach enables direct derivation of bending moduli in different contexts, while dissecting the contributing factors and underlying mechanisms, be it lipid tilt, changes in chain flexibility, or hydrophobic hydration. Our results are in excellent agreement with giant-vesicle measurements, confirming that cholesterol effects are lipid-specific and explaining why certain experiments probing the nanometer scale diverge. In summary, we demonstrate that quantitative structure-mechanics relationships can now be established for mixed lipid membranes, paving the way to addressing open central questions in cell membrane mechanics.

## Introduction

All biological membranes resist deformations of their intrinsic shape. A membrane-bound protein can however reshape the surrounding bilayer, sometimes strikingly, because the free-energy cost of membrane bending is offset by free-energy gains resulting from adequate solvation of the protein surface [1]. In other words, lipid bilayers change shape around proteins to avoid large energetic penalties due to dehydration of ionized surface residues or exposure of large hydrophobic clusters to water [2, 3, 4, 5, 6, 7, 8]. This kind of balance between competing energetic contributions is not uncommon, and governs many other processes in membrane physiology, including ligand-induced allostery, ion permeation, etc. However, while atomic-resolution perspectives have become the state-of-the art in experimental and theoretical analyses of both protein structural dynamics and solvation energetics, membrane morphology is still often conceptualized as a continuum-mechanics problem. While insightful in several cases [9, 10, 11], the shortcomings of this perspective become quickly apparent for non-homogenous lipid bilayers, which are the norm in biological cells. And so, deceptively simple fundamental questions, such as how the chemical makeup of a bilayer dictates its intrinsic bending energetics, remain largely unresolved.

A recent controversy regarding the stiffening effect of cholesterol highlights this challenge. Cholesterol is known to be enriched in animal cell membranes, and had been thought to universally enhance their rigidity by dampening the movement of other lipids [12, 13]. If so, generating curvature in cellular membranes would be more costly than in the synthetic bilayers often examined in laboratory conditions, and potentially entail distinct mechanisms. However, while the predicted stiffening effect was confirmed for bilayers of partially or fully saturated lipids [14, 15], cholesterol was observed to have little or no effect on unsaturated lipids like DOPC, either examined with X-ray diffraction [15], tube aspiration [16, 17] and electro-deformation [18]. This lipid-type specificity would have interesting biological implications. For example, unsaturated lipids might localize at the periphery of domains enriched in saturated lipids [19] to mitigate stiffening and help preserve the overall membrane plasticity wherever needed. While appealing, however, this concept has been disputed by a recent study of the bending rigidity of DOPC membranes, in the presence and absence of cholesterol, using neutron-spin echo (NSE) and nuclear magnetic resonance (NMR) [20]. In stark contrast to the preceding studies, this analysis concluded that the stiffening effect of cholesterol is likely universal for all lipid bilayers, a notion that quickly became the subject of debate [21, 22]. It could be argued that in no small part this controversy stems from the fact that bending moduli are not measured directly but inferred from other quantities, and that models used to establish that inference tend to neglect the complex nature of the lipid bilayer, or examine its structure and dynamics without an actual morphological perturbation.

In this work, we showcase an all-atom molecular simulation methodology we recently developed [23] to address this kind of challenge. This methodology, which we refer to as Multi-Map, provides a means to directly quantify free-energies of bending for lipid bilayers of any chemical makeup, including model synthetic bilayers with or without cholesterol. This approach mimics the action of proteins (or of laboratory manipulations) that sustain curvature over long time scales, and it provides atomically-resolved insights into the structure and dynamics of the membrane. Our results unequivocally support the notion that the stiffening effect of cholesterol is nonuniversal [15, 16, 17, 18], and that it is specifically dependent on the degree of lipid-chain unsaturation. Importantly, our results also reveal the mechanism by which lipid unsaturation enables the membrane to preserve flexibility despite the increased density and molecular order that cholesterol invariably induces. These insights permit us to explain the divergence between the abovementioned studies of unsaturated membranes. In conclusion, we posit that this emerging computational methodology, in combination with suitable experimental approaches, will facilitate a clearer understanding of the molecular mechanisms by which cells and organelles shape the morphology of lipid membranes.

## Results

### Energetics of applied curvature vary with composition and length scale.

Atomistic models of hydrated bilayers were prepared for the following compositions: pure POPC, pure DOPC, and 70:30 mixtures of either POPC or DOPC with cholesterol (CHOL), hereafter indicated as POPC/CHOL and DOPC/CHOL. POPC and DOPC lipids were chosen because they have very similar bending moduli and radii of preferred curvature [24, 25]. A 30% cholesterol mole fraction represents typical concentrations in eukaryotic membranes [26], and approaches the highest concentration that is still soluble in physiological phospholipids [27, 28]. This concentration has also been shown to induce significant changes in molecular properties [20] and membrane mechanics [14, 15].

For each composition, three bilayers of different sizes were prepared, including 200, 800 and 1800 lipid molecules for a total of 12 membrane models. These correspond to approximately 80×80, 160×160 and 240×240 Å^2^ in lateral dimensions for the pure bilayers, and 70×70, 140×140 and 210×210 Å^2^ for the mixed bilayers. All bilayers were simulated in atomistic detail and explicit solvent over multiple microseconds at room temperature and pressure using two different sampling methods (Table S1).

**Figure 1:**
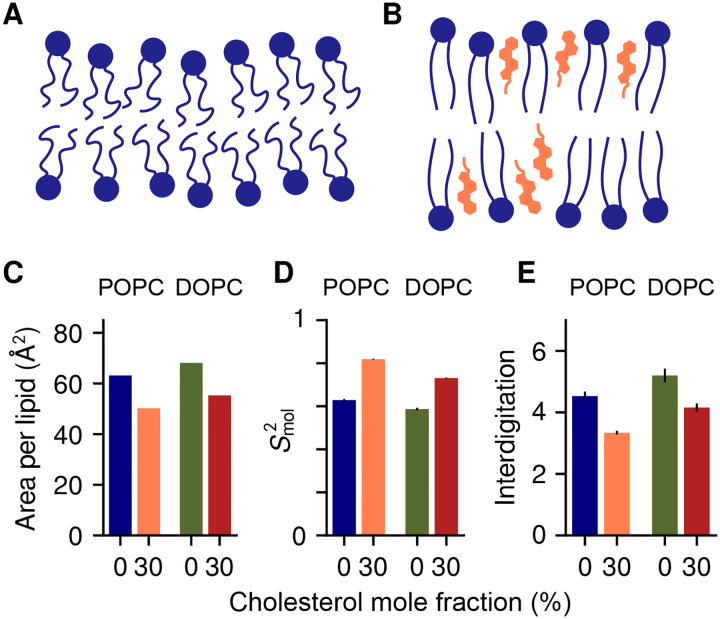
Cholesterol increases molecular order. *(A, B)* Schematic visualization of the established effects of cholesterol on phospholipids. *(C)* Area per phospholipid molecule in POPC (blue), POPC/CHOL (orange), DOPC (green) and DOPC/CHOL (red). *(D)* Orientational order parameter $S_{\text{mol}}^{2}$ of lipid molecules. *(E)* Degree of interdigitation between leaflets (in number of acyl chain bonds).

**Figure 2:**
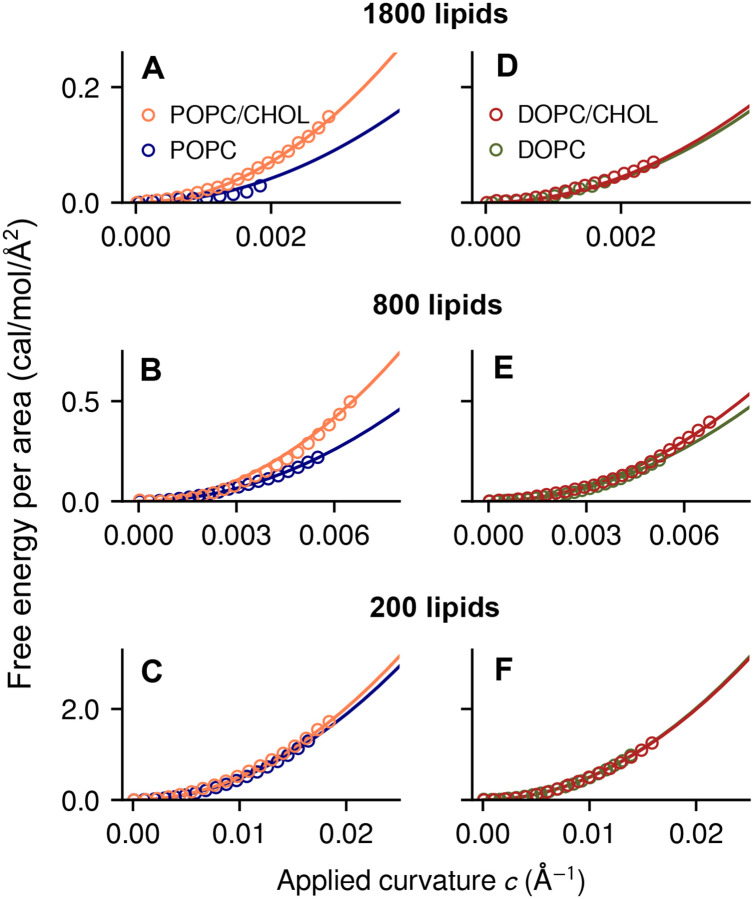
Thermodynamics of lipid bilayers under applied curvature. Shown are PMFs of POPC (blue) and POPC/CHOL bilayers (orange), containing 1800 *(A)*, 800 *(B)* and 200 lipid molecules *(C)*. Panels *(D)* through *(F)* show the equivalent results for DOPC (green) and DOPC/CHOL (red). All PMFs are expressed in units of free-energy per area as a function of curvature. Symbols indicate computed values, and solid lines are quadratic fit curves.

First, we simulated the bilayers without applying any extrinsic forces, to examine their spontaneous fluctuations. This analysis confirms that cholesterol increases the molecular order of the lipid bilayer, for both POPC and DOPC (Fig. 1). From these trajectories, we then extracted snapshots with increasingly high curvature (see Methods), and used the Multi-Map enhanced-sampling method to quantify the energetics of these curved states [23]. In this approach, a collective variable (or reaction coordinate) that reflects the magnitude of membrane curvature [23] is gradually biased to explore a range of values, by applying a series of shifting biasing potentials following the umbrella-sampling protocol [29]. It is important to note that while this bias fosters an average shape in the membrane, it does not influence its fluctuations around that shape, nor does it impose one or other mechanism of bending at the molecular level. Through post-processing of these enhanced simulations, we then derived for each bilayer the potentials of mean force (PMFs) as a function of the applied curvature *c*, shown in Fig. 2 in units of free energy per area.

These data demonstrate that the free-energy cost of deformation of a POPC/CHOL bilayer is significantly higher than that of a POPC bilayer of comparable size (Fig. 2A,B), i.e. cholesterol has a substantial stiffening effect on POPC. By contrast, the free-energy landscapes for DOPC and DOPC/CHOL are nearly identical (Fig. 2D–F), i.e. cholesterol has a minimal influence on the flexibility of the membrane despite its effect on molecular order.

It is worth noting that, notwithstanding the computational cost of these calculations, the PMFs shown in Fig. 2 have very low statistical error, and thus those curves are faithful representations of the free-energy cost of membrane bending at the corresponding length scales (for the particular simulation forcefield used). However, the derivation of the bending modulus *k*_*c*_ from the PMFs requires further analysis of the simulation data, because the free-energy cost of curvature is often mitigated by a change in molecular orientation [30, 31, 32, 25, 33]. It is mainly for this reason that the magnitude of the cholesterol effect on POPC varies with bilayer size, becoming very small for the smallest bilayers, *L* < 80 Å (Fig. 2C). This problem is addressed in the next section, by quantifying simultaneously the energetics of membrane curvature and lipid tilt.

### Determination of bending and tilt moduli.

The Helfrich-Canham theory [34, 35] describes the free-energy density *f*_HC_ of bending a membrane through a continuum description of the bilayer plane. For a symmetric and periodic bilayer the theory relies on a single parameter, the bending modulus *k*_*c*_, and has the following expression:

(1) $$f_{\text{HC}}[h]=\frac{1}{2}k_{c}{\left(\nabla \cdot \text{N}\right)}^{2}$$

where *h*(*x, y*) is a two-dimensional function that maps the difference between the shape of the deformed bilayer and a flat shape. *f*_HC_ depends directly on the second derivatives of *h*, or equivalently the first derivatives of the vector perpendicular to the membrane midplane **N** =(*∂*_*x*_*h, ∂*_*y*_*h,* −1), save for normalization. The Helfrich-Canham model therefore implicitly assumes that, on average, all lipid molecules remain parallel to the normal vector **N**.

**Figure 3:**
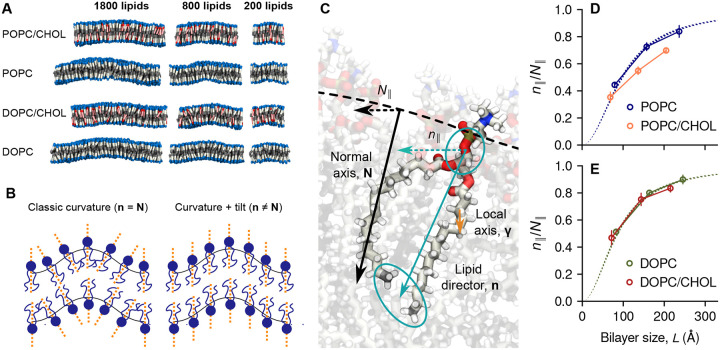
Structures of curved bilayers are described by their balance of curvature and tilt, eq. 2 [30]. *(A)* Atomistic snapshots of bilayers under applied curvature, with phospholipid molecules (blue, gray) and cholesterol (red, pink) visualized as rods whose endpoints define the lipid orientation vectors **n**_*i*_. *(B)* Schematic of a bilayer’s structure when either term of eq. 2 dominates the other. *(C)* Illustration of the membrane normal vector, **N**, the lipid orientation vector **n**, and the local chain vector *γ* (analyzed in Fig. 6). The ratio *n*_∥_*/N*_∥_ measures the relative weight of the curvature energy; *n*_∥_*/N*_∥_ = 1 is the Helfrich-Canham theoretical limit of long wavelength. *(D,E)* Mean values of *n*_∥_*/N*_∥_ for POPC (blue), POPC/CHOL (orange), DOPC (green) and DOPC/CHOL (red); error bars represent standard errors. Dashed lines show the theoretical predictions for DOPC and POPC using published parameters.

While this assumption is plausible for ‘macroscopic’ long-wavelength deformations, it has later been recognized that bending at ‘microscopic’ short wavelengths also entails changes in lipid orientation. A more accurate free-energy functional is the following [30]:

(2) $$f_{\text{HK}}[h,\text{n}]=\frac{1}{2}k_{c}{\left(\nabla \cdot \text{n}\right)}^{2}+\frac{1}{2}k_{t}|\text{n}-\text{N}{|}^{2}$$

where **n** maps the lipid orientation across the membrane and *k*_*t*_ is referred to as the lipid tilt modulus. Note that while the first term in Eq. 2 is very similar to Eq. 1, **n** varies more smoothly than **N** at short wavelengths (< 100 Å, Fig. 3B) and thus the free-energy cost of bending according to Eq. 2 is significantly smaller than that predicted by the Helfrich-Canham model (Eq. 1). The spectral density of fluctuations generated by Eq. 2 has been supported by many simulations and experiments [36, 37, 31, 25, 33].

A common approach to estimate *k*_*c*_ and *k*_*t*_ is to examine the spontaneous fluctuations of a membrane, either by simulation or experiment: this approach is physically correct but can prove challenging. Because membrane fluctuations are transient, **n** and **N** are difficult to resolve in both position and time, requiring substantial averaging. This challenge is greater when the intrinsic bilayer shape is flat, as **n** and **N** fluctuate around the same fixed direction, and only their fluctuations carry physical information. By contrast, evaluation of n and N can be more accurate under applied curvature, as each will vary significantly across the bilayer, to an extent that can be predicted by theory.

For example, for a periodic sinusoid along the *x*-axis, which is the bending mode induced in our simulations, the three components of **N** and their derivatives can be calculated easily: the only longitudinal component is *N*_*x*_, hereafter labeled as *N*_∥_. Minimizing Eq. 2 with respect to the corresponding orientation component, *n*_∥_, the following expression is obtained:

(3) $$\frac{n_{\|}}{N_{\|}}={\left(1+\frac{k_{c}q^{2}}{k_{t}}\right)}^{-1}$$

where *q* = 2*π/L* and *L* is the bilayer size. Note that because *N*_∥_ and *n*_∥_ are constant along the *y* axis, the “twist” term ∇×(**n**−**N**) [30, 31, 33] can be neglected safely and thus was not included here for clarity. Splay-tilt coupling [38] was similarly neglected because it is thought to be significant only at very high *q* [33].

Based on the same trajectory data used for the derivation of PMF curves (Fig. 2) we calculated the ratio *n*_∥_*/N*_∥_ for each bilayer using linear regression (Fig. S1), consistent with the underlying assumption of linear response [30]. For pure POPC and DOPC, we find that the simulation data and Eq. 3 are in excellent agreement (Fig. 3D and E) using published values of *k*_*c*_*/k*_*t*_ [24] without any fitting. Eq. 3 also describes the data very well for POPC/CHOL and DOPC/CHOL, using as the only fitting parameter the unknown ratio *k*_*c*_*/k*_*t*_ (Fig. S2).

**Figure 4:**
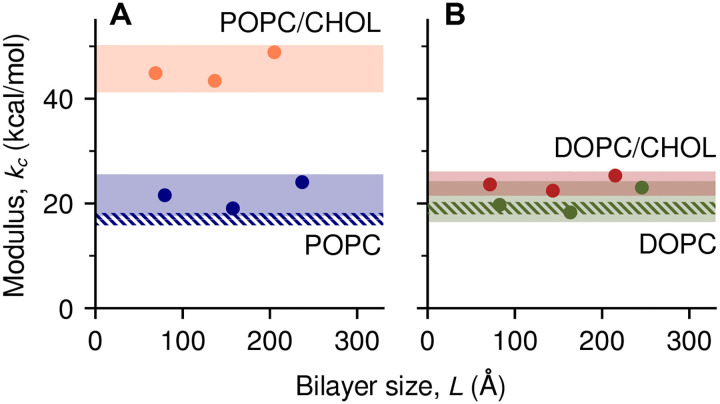
Macroscopic bending moduli *k*_*c*_ of POPC and POPC/CHOL 7:3 *(A)*, DOPC and DOPC/CHOL 7:3 *(B)*, obtained from computed PMFs under applied curvature. Shaded bands show the estimated 95% CIs of *k*_*c*_, and dashed bands the 95% CIs of *k*_*c*_ from tilt fluctuations of pure lipids [24].

As shown in Fig. 4 and Table 1, the results of this analysis show that *k*_*c*_ increases by a factor of 2.1 ± 0.2 when POPC and POPC/CHOL are compared (Fig. 4A), confirming cholesterol has a significant stiffening effect for this type of lipid. Our estimate is indeed in excellent agreement with the 2.3-fold increase measured by tube aspiration [14]. By contrast, the relative change in *k*_*c*_ between DOPC and DOPC/CHOL is 1.1 ± 0.1 (Fig. 4B), consistent with measurements that indicate no stiffening [15, 16, 17, 18]. Although those experiments probed “macroscopic” wavelengths, our results now show that the effect of cholesterol is also minimal at length scales comparable to the membrane thickness (*L* < 100 Å).

In summary, our results unequivocally demonstrate that the stiffening effect of cholesterol is nonuniversal – and more specifically, that it is negligible for an unsaturated lipid like DOPC. Evidently, this conclusion rests on the two key elements of our theoretical analysis, namely the atomic-resolution force field used in the simulations [39], and the curvature-tilt energy functional described in Eq. 2 [30]. Nonetheless, the validity of both is well documented [24, 36, 37, 31, 25, 33], and so it is difficult to envisage how the opposite conclusion in regard to the stiffening effect of cholesterol could emerge from an alternative analysis of simulation data.

### Location of unsaturated bonds is key for curvature generation.

Although continuum theory can be used to calculate energy landscapes of membranes when empirical parameters such as *k*_*c*_ and *k*_*t*_ are known or assumed, it cannot explain how those energy landscapes emerge. This explanation can only be obtained by examining the internal molecular structure of curved bilayers and the precise configuration of their constituent lipids. To that end, in addition to the the change in molecular orientation discussed above, we used our trajectory data to examine other “microscopic” properties, such as membrane thickness, local composition, as well as hydration and structural changes in the hydrocarbon chains. All of these properties fluctuate on time scales shorter than microseconds, and therefore our trajectories yield excellent statistics.

**Figure 5:**
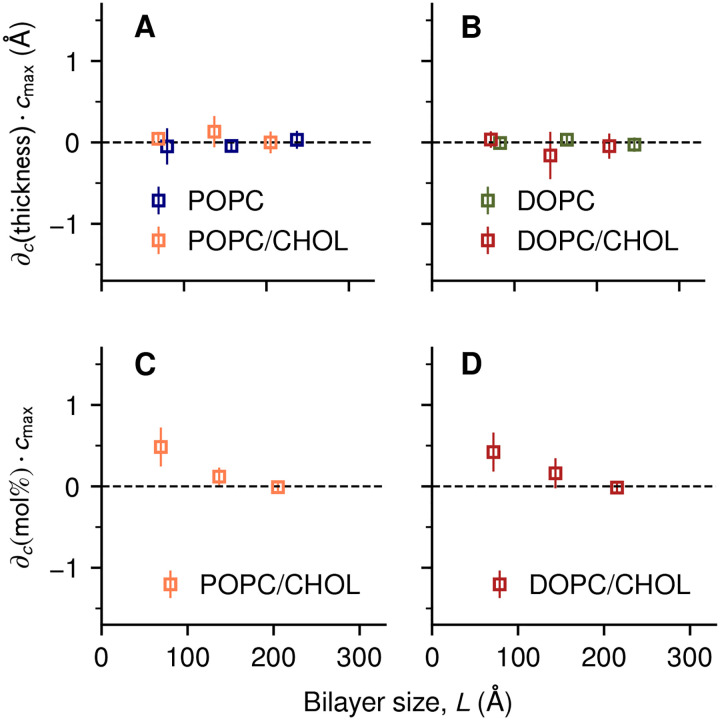
Variations in bilayer thickness and in local composition do not explain bending energetics. Derivatives of each property *X* with respect to curvature, *∂*_*c*_(*X*), were estimated by linear regression; for ease of comparison, the resulting slopes were multiplied by the largest applied curvature *c*_max_. *(A)* Changes in thickness (in Å units) for POPC and POPC/CHOL and *(B)* for DOPC and DOPC/CHOL bilayers. *(C)* Changes in cholesterol concentration (in mol% units) in POPC/CHOL and *(D)* for DOPC/CHOL bilayers. Error bars represent standard errors.

No significant correlations were observed between applied curvature and local bilayer thickness for any bilayer size (Fig. 5A and B). For the smallest of the mixed bilayers (*L* < 100 Å), a small redistribution of cholesterol molecules was observed, i.e. a < 0.5 mol% increase in local concentration at locations with the highest curvature (*c* ≈ 0.02 Å ^−1^, Fig. 5C,D). While consistent with cholesterol’s preference for concave membrane surfaces, this enrichment is localized within very short distances and is similarly small between POPC/CHOL and DOPC/CHOL. Therefore, cholesterol enrichment is unlikely to explain why the energy landscapes of the largest bilayers differ (Fig. 2A,B). That is, cholesterol may favor certain morphologies at constant-curvature conditions (e.g. in a vesicle), but such preference does not seem to overcome the entropic cost of demixing in a bilayer with varying curvature. Conversely, water exposure of the acyl chains (hydrophobic hydration) was found to be significantly correlated with curvature, but only for pure lipids and not for their cholesterol mixtures (Fig. S3).

Atomic-level differences between DOPC and POPC were however revealed by measuring the extent to which curvature affects the orientation of individual acyl-chain segments. This analysis entails computing the vector formed by two carbon atoms spaced by two chemical bonds:

(4) $$\gamma (i)=\left({\text{x}}_{i+1}-{\text{x}}_{i-1}\right)$$

Similar to the analysis associated with Eq. 3, the longitudinal component of this vector, *γ*_∥_(*i*), was evaluated and the ratio *γ*_∥_(*i*)*/N*_∥_ quantified through regression. The result was then normalized against *n*_∥_*/N*_∥_, to analyze the movements of the *i*-th chain segment relative to all others in the same lipid chain. Higher values of *γ*_∥_(*i*)*/N*_∥_ indicate a higher flexibility in the *i*-th chain segment.

**Figure 6:**
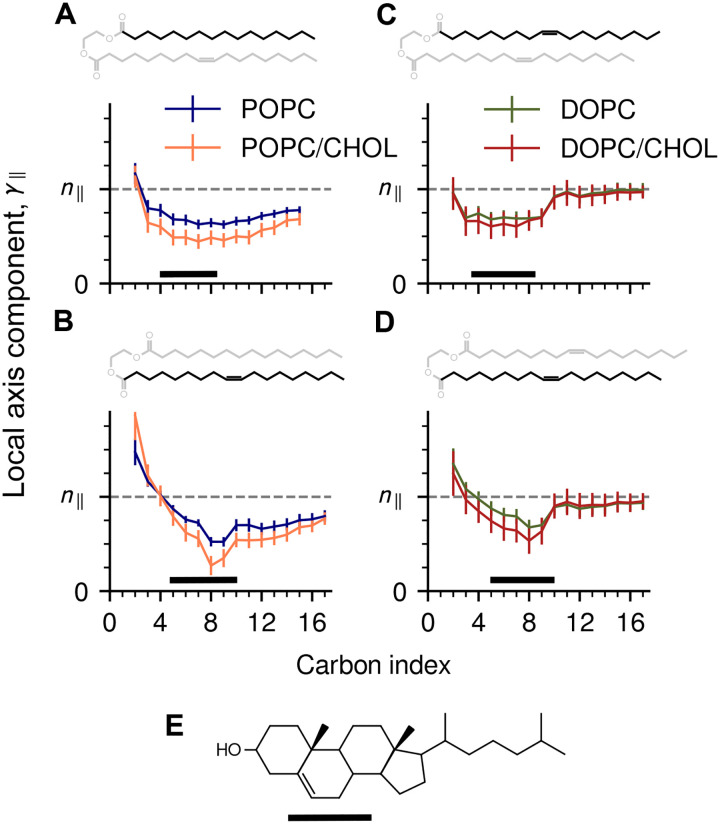
Concurrent and strategically located unsaturated bonds confer the ability to adapt to curvature. Shown are mean values of the longitudinal component *γ*_∥_ of the local chain axis (Fig. 3C), normalized against the lipid molecule’s orientation *n*_∥_, shown as dashed lines. *(A)* Values of *γ*_∥_ for the *sn*-1 chain and *(B) sn*-2 chain of POPC (blue) and POPC/CHOL (orange). *(C,D)* Equivalent results for DOPC (green) and DOPC/CHOL (red). In each panel the chemical structure of the acyl chain is highlighted. Black bars indicate the central 50% of the distribution of the sterol group, shown in *(E)* for reference.

The results of this analysis are shown in Fig. 6. In the absence of cholesterol, *γ*_∥_(*i*)*/N*_∥_ is highest for the chain segments that are most proximal to the head groups (*i <* 3) and decays rapidly over a distance shorter than the chain persistence length (≈ 6 Å). Cholesterol lowers the value of *γ*_∥_(*i*)*/N*_∥_ even further, with the largest changes observed in the saturated *sn*-1 chain of POPC (Fig. 6A), and near the sterol group in general (4 ≤ *i* ≤ 10). It is outside of this region, however, that DOPC and POPC respond to curvature in entirely different ways.

In DOPC, segments of 8 or more carbons in each chain pivot relatively freely, manifested as *γ*_∥_ ≃ *n*_∥_, and the “hinge points” of this motion are marked by the unsaturated bonds (Fig. 6C,D). Strikingly, the ability of these segments to move is completely unaffected by cholesterol, explaining why the free-energy of membrane bending does not increase for DOPC/CHOL.

The dynamics is entirely different for POPC: the unsaturated *sn*-2 chain has little effect on the saturated *sn*-1 chain (Fig. 6A,B), which remains relatively rigid and shows limited ability to rotate. When cholesterol is added, the rigidity of the *sn*-1 chain increases further, explaining the higher free-energy cost needed to generate the same curvature.

### Fluctuations of single lipid molecules are universally dampened by cholesterol.

To rationalize the apparent discrepancy between measurements at “macroscopic” length scales [15, 16, 17, 18] and those at “microscopic” scales [20], it is instructive to examine the role of fluctuations, because most of these experiments were carried out without applying curvature. Dynamics were analyzed at three length and time scales: *(i)* atomic-scale fluctuations of the acyl chains (≤ 0.1 ns), *(ii)* reorientation of lipid molecules (1–10 ns), and *(iii)* collective membrane-bending fluctuations (> 100 ns). Among the applicable techniques, X-ray diffraction and NSE typically measure only the third type [25, 20], NMR may span two types concurrently [40], and MD simulation can nowadays access all three.

There is consensus over the fact that when cholesterol is mixed with any phospholipid, it significantly decreases its flexibility and increases its alignment with the membrane normal, as illustrated in Fig. 1A,B. Both effects are indeed observed in our simulations concurrently, through changes in the area per lipid molecule (Fig. 1C) and atomic order parameters (Fig. S4). Consistent with earlier analyses [41], the change is higher in saturated chains (*sn*-1 chain of POPC) than in unsaturated chains (DOPC and *sn*-2 chain of POPC).

Atomic and molecular fluctuations can also be analyzed separately. For example, while atomic order parameters also depend on the overall molecular orientation, the conformational entropy of the acyl chains does not (Fig. S5). By estimating the loss of entropy per molecule as the sum of contributions from individual torsional angles, the resulting free-energy change upon addition of cholesterol is −*T*Δ*S* = 1.05 kcal/mol for POPC, and 0.44 kcal/mol for DOPC (Fig. S5). While this result suggests a measurable difference in how the two lipids responds to cholesterol, it is also unrelated to curvature and thus insufficient to explain differences in membrane bending.

Separate from atomic fluctuations, the orientational order of lipid molecules can be quantified through the following auto-correlation function (ACF):

(5) $${\langle P_{2}\left({\text{n}}_{i}\left(t_{0}\right)\cdot {\text{n}}_{i}\left(t_{0}+t\right)\right)\rangle }_{t_{0},i}$$

where **n**_*i*_ is the orientation vector of the *i*-th lipid molecule, *t*_0_ and *t* are time intervals, and *P*_2_(*x*) = (3×^2^−1)*/*2. Lipid orientation fluctuations are much faster than membrane curvature fluctuations [31, 24]: therefore, Eq. 5 has a relatively well defined plateau near *t* = 100 ns, here indicated as $S_{\text{mol}}^{2}$. Cholesterol causes a significant increase in $S_{\text{mol}}^{2}$ for both POPC and DOPC (Fig. 1D). The specific values of $S_{\text{mol}}^{2}$ for DOPC and DOPC/CHOL (0.59 and 0.73, respectively) are also in close agreement with previous sub-*μ*s simulations [20], confirming that $S_{\text{mol}}^{2}$ is insensitive to membrane curvature.

### Collective membrane-bending fluctuations confirm previous results.

Shape fluctuations of the membrane midplane were computed at each time frame from the centers of mass of each lipid molecules minus the bilayer’s mean vertical position, *h*(*x*_*i*_*, y*_*i*_)=(*z*_*i*_ − 〈*z*〉). The 2D Fourier transform of *h*, indicated as *h*_**q**_, was computed over all wave vectors with wavenumber |**q**| < 0.2 Å^−1^, which is appropriate for atomistic simulations [33]. Values of |*h*_**q**_|^2^ were averaged over trajectory frames with times *t >* 0.5 *μ*s, and data points with equal |**q**| combined. Based on Eq. 2, the following expression was used to model bending fluctuations spectra [30, 31, 25, 33]:

(6) $$\langle {\left|h_{\text{q}}\right|}^{2}\rangle =k_{\text{B}}T\left(\frac{1}{k_{c}q^{4}}+\frac{1}{k_{t}q^{2}}\right)$$

For clarity and completeness, we also mention briefly a more recent method [31], which analyzes the fluctuations of the lipid tilt field (**n**−**N**) uniquely accessible to MD simulation. (NMR also measures the fluctuations of **n**, but they are aggregated with those of **N** as well as atomic movements [40].) This tilt-fluctuations method [31] can be more precise than the bending-fluctuations method (Eq. 6), but it has not yet been validated on mixed-composition bilayers and was not used here.

**Figure 7:**
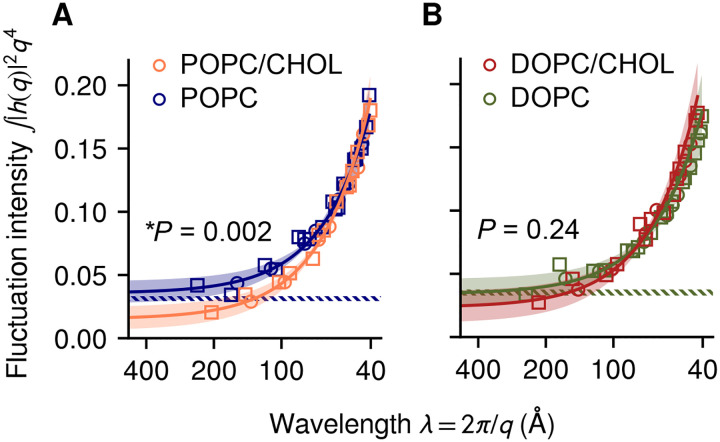
Spectral analysis of bending fluctuations for POPC and POPC/CHOL bilayers *(A)* and DOPC and DOPC/CHOL bilayers *(B)*. Solid lines and shaded bands show best fits using eq. 6 and the 95% confidence intervals (CIs) around them; *P*-values for comparisons of *k*_*c*_ with and without cholesterol are also reported. Dashed bands show the 95% CIs for POPC and DOPC under the *q*^−4^ long-wavelength Helfrich-Canham theory [34, 35] using published values of *k*_*c*_ [24].

The spectral analysis of bending fluctuations is visualized in Fig. 7 and its results listed in Table S2. The resulting bending moduli *k*_*c*_ for POPC and DOPC have reasonable uncertainty (~15%) and agree well with values from the tilt fluctuation method or “flicker” spectroscopy [31, 24], but are slightly larger than those from X-ray diffraction [25]. The latter discrepancy has been discussed previously but not yet understood [24, 25]: it has been speculated that the close spacing of multi-lamellar stacks used for X-ray diffraction introduces a favorable coupling between bilayers [24]. Nonetheless, all methods agree that POPC and DOPC have nearly identical *k*_*c*_ and *k*_*t*_.

For POPC/CHOL and DOPC/CHOL, statistical uncertainty is significantly higher (~35%, Table S2), possibly due to the higher viscosity limiting sampling despite the longer simulation times (Table S1). Only one significant difference is detected, between the bending moduli *k*_*c*_ of POPC and POPC/CHOL (**P = 0.002), consistent with the 2.3-fold increase measured by tube aspiration [14]. It was not possible to detect significant changes between DOPC and DOPC/CHOL.

## Discussion

To examine opposite claims in regard to the universality of cholesterol stiffening of lipid bilayers, resulting from distinct experimental approaches [21, 22], we used a recently developed methodology [23] to compute bending free-energy curves for atomically-detailed bilayers of POPC, DOPC and their mixtures with 30 mol% cholesterol at length scales relevant in molecular physiology (~100–250 Å). Our results clearly show that while POPC and DOPC have virtually identical mechanical properties, the addition of 30 mol% cholesterol increases the bending modulus *k*_*c*_ of POPC over two-fold but does not affect DOPC. These results are thus in line with a body of experimental evidence that supports the notion that the stiffening effect of cholesterol is lipid-type specific, rather than universal [14, 15, 16, 17, 18].

Because our computational methodology entails simulation of all-atom trajectories, it not only quantifies the conformational energetics of the membrane but also explains its origins with molecular resolution. Our trajectory data show that while POPC and DOPC bilayers appear comparable when described by macroscopic parameters such as bending modulus or preferred curvature, they differ significantly when examined at the atomic level, and these differences in turn explain why cholesterol has a distinct effect in each case. Specifically, we observe that the unsaturated bonds cause both chains in DOPC to change orientation much more dynamically than the chains in POPC, and this configurational freedom is not impacted by cholesterol owing to the precise location of these bonds (Fig. 6). Interestingly, this location is highly conserved in mono-unsaturated lipid chains [42, 41], suggesting that this physical property serves important biological functions.

The DOPC and DOPC/CHOL results here shown are in apparent disagreement with recent NMR, NSE and MD data that suggest strong stiffening [20]. However, it is important to note that the techniques used in that work do not measure directly the energy cost of membrane bending, but rather infer it from molecular-scale fluctuations. Therefore, the conclusions of that paper hinge upon the theoretical models and approximations used. For example, Chakraborty *et al* [20] used the Helfrich-Canham theory [34, 35] to parameterize membrane bending energetics. Although effective for long wavelengths, this theory is insufficient to describe membrane fluctuations at the nanometer scale [36, 37, 31, 25, 33]. The magnitude of this approximation can be seen in Fig. 7, where membrane bending fluctuations significantly exceed the Helfrich-Canham prediction, to an extent that depends both on length scale and lipid composition. Other approximations specific to each technique used in that study [20] are briefly discussed as follows.

The NSE method relies on experimental detection of the relaxation rates of collective membrane fluctuations over tens of Å and hundreds of nanoseconds [43, 20]. This ability grants access to unique insights on the structural dynamics of mixed membranes: for example, NSE has recently demonstrated that binary mixtures of saturated lipids (DMPC/DSPC) have non-additive properties [44]. However, because NSE is sensitive to many kinds of membrane fluctuations, theoretical models are required to isolate each contribution. Specifically, bending fluctuations are estimated by analyzing NSE-measured decay rates as a function of wavelength [45, 46]. Although the underlying theory acknowledges the existence of “fast” and “slow” exponential decays [47], single exponentials are assumed due to the short time span of NSE experiments [46]. This assumption is tested here by examining the time auto-correlation functions of the Fourier coefficients *h*_**q**_, whose variances |*h*_**q**_|^2^ were previously analyzed in Fig. 7. We find that *h*_**q**_ decays as a single exponential only for pure POPC or pure DOPC, but not for POPC/CHOL and DOPC/CHOL (Fig. S6A–H). The source of this deviation is unclear: however, its presence calls for a reassessment of the procedure used to extract bending moduli from NSE data [20], e.g. by accounting explicitly for multiple dissipative motions in the membrane. Because interleaflet friction can now be measured directly with reasonable accuracy [48], future NSE studies might leverage other experiments to reduce the number of theoretical assumptions.

Unlike NSE, NMR experiments track membrane dynamics by measuring the orientational order of specific chemical bonds in the lipid chains. Atomic, molecular and collective fluctuations all contribute together, and are analyzed using a kinetic theory that correlates order parameters with relaxation rates for each bond [40]. Specifically, the extraction of a bending modulus *k*_*c*_ from NMR data was done by summarizing collective membrane fluctuations through a fixed “slow” order parameter, *S*_s_ [40, 20, 49]. With this approximation, the relative magnitudes of the different types of fluctuations are imposed by the model, rather than being measured for the specific membrane.

Lastly, atomistic MD simulations of bilayers at rest offer the means to quantify each type of fluctuation independently. In practice, statistical sampling of bending fluctuations (i.e. collective) can be limited in length and time scale [33], which has prompted some researchers to infer those fluctuations from molecular descriptors. In Chakrarborty *et al* [20], the fluctuations of the “splay” angle between pairs of neighboring lipid molecules were analyzed [50]. We reproduce those results in Fig. S7, but did not use them to derive macroscopic moduli. One of the underlying assumptions of that approach is that the fluctuations of the lipid tilt vector (**n**−**N**) are decoupled from those of the normal vector, **N** [50]. This approximation is reasonable only for larger bilayers, and requires adopting a moving frame of reference to account for the fluctuations of **N**, without assuming it constant over the bilayer [31, 24]. This problem is innate in the analysis of membrane fluctuations, and was avoided here by examining changes in **n** and **N** that significantly exceed those fluctuations.

We conclude this discussion by underscoring an important result specific to the shortest length scales considered in our simulations, namely *L* < 100 Å. In this case, the mechanism of curvature generation remains unchanged upon addition of cholesterol, and there is little or no stiffening effect for *either* DOPC or POPC (Fig. 2). This observation suggests that individual membrane proteins interact with a mechanically invariant environment even when the neighboring concentration of cholesterol fluctuates. In other words, while cholesterol can impact membrane remodeling at scales comparable to lipid domains [19], it would not disrupt the activity of individual membrane proteins whose mechanisms entail local deformations of the membrane [4, 5]. Quantitatively testing this hypothesis will prove essential for establishing how protein biochemistry dictates membrane morphology.

## Methods

### Unbiased simulations and analysis

Initial atomistic models of hydrated lipid bilayers were obtained by replicating pre-equilibrated configurations from an earlier study [23]. The water buffer that separates the periodic images of each bilayer is ~ 70 Å thick, representing a water content about four times higher than in the multilamellar samples used for X-ray diffraction [25]. Interatomic forces were represented via the CHARMM36 and TIP3P force fields [39, 51]. Unbiased MD simulations were performed with NAMD [52], using a 12 Å cut-off for Lennard-Jones forces, covalent bonds involving hydrogens constrained, a 2 fs integration step and 300 K and 1 atm target temperature and pressure. Lipid orientation vectors **n**_*i*_ were defined consistent with earlier work [24, 33]. Coordinates were recorded every 20 ps and then used for analyses, most of which were implemented via Python scripts based on MDAnalysis [53]; lipid interdigitation was computed using MOSAICS [54]. Simulation snapshots were rendered with VMD [55].

### Potentials of mean force (PMF) computations

One-dimensional density profiles of the lipids’ phosphate atoms were used to construct three-dimensional density maps, one for each leaflet, in a state of zero curvature. Sinusoidal deformations given by cos(2*πx/L*_*x*_) were then applied to each leaflet’s map to produce additional maps *φ*_*k*_(x) consistent with curved bilayer states. All maps were then included in the definition of a Multi-Map collective variable [23]:

(7) $$\xi \left({\text{x}}_{1},\ldots {\text{x}}_{N}\right)=\sum_{k=1}^{K}\xi_{k}\sum_{i=1}^{K}\phi_{k}\left({\text{x}}_{i}\right)$$

where **x**_*i*_ are coordinates of the phosphate atoms and *ξ*_*k*_ are constants measuring the amount of curvature in each map. Given the choice of sinusoidal shape to generate the curved maps, *ξ* is directly proportional to $\text{Re}\left(h_{{\text{q}}_{\left(1,0\right)}}\right)$ [56, 23]. More complex shapes are also supported by the method [23, 4], but here a sinusoid allows for simpler theoretical expressions of the relevant vector fields (eq. 3). Cholesterol molecules were not included in the definition of *ξ* due to the possibility of their migration between leaflets; it was later verified that the lateral distribution of cholesterol is not significantly correlated with curvature for the compositions used (Fig. 5).

Values of *ξ* from each unbiased trajectory were collected into histograms with spacing ≈ 0.2 times the standard deviation of *ξ*. Random frames from each histogram bin were used to initialize umbrella-sampling windows [29]. Using uncorrelated initial snapshots improves statistical sampling compared to previous computations with Multi-Map [23]. Umbrella-sampling windows were simulated concurrently using NAMD and the Colvars module [57], with harmonic restraints on *ξ* of force constant 0.6 kcal/mol for ≈ 1 *μ*s (Table S1). Potentials of mean force (PMFs) were extracted using the WHAM [58] and FCAM [59] methods, with no significant differences between the two. To maximize sampling of the slower-moving cholesterol-rich bilayers, the 1800-lipid POPC and DOPC bilayers were simulated for shorter times to examine their structural changes, but their PMFs were extracted from unbiased histograms.

## Supplementary Material

Supplement 1

## Figures and Tables

**Table 1:** Bending and tilt moduli for pure and mixed bilayers (means and 95% confidence intervals).

	*k*_*c*_ (kcal/mol)	*k*_*t*_ (kcal/mol/Å^2^)
POPC	21.6 [17.6, 25.6]	0.104 [0.085, 0.124]
POPC/CHOL	45.7 [41.2, 50.2]	0.111 [0.100, 0.122]
DOPC	20.9 [17.1, 24.7]	0.114 [0.094, 0.134]
DOPC/CHOL	23.8 [21.5, 26.1]	0.134 [0.121, 0.147]

## Significance

Quantifying the energy necessary to remodel biological membranes is an essential step in understanding cell physiology. However, membrane bending involves both collective and atomic-level movements that are difficult to detect simultaneously. Here, atomically-detailed simulations fill a gap between those measurements. The results address a controversy that had recently emerged about the stiffness of cholesterol-rich membranes, which are ubiquitous in animal cells. The reason behind the surprising behavior of unsaturated lipids like DOPC is also revealed. Clearer connections are established between lipid chemistry and membrane mechanics, providing a more solid basis for future research on phenomena like cell trafficking or viral infection.
