# Supplementary material for: Membrane free-energy landscapes derived from atomistic dynamics explain nonuniversal cholesterol-induced stiffening
Source: bioRxiv. 2023 Feb 3:2023.02.02.525347. Preprint. [Version 1] doi: 10.1101/2023.02.02.525347 (PMC9915699; doi:10.1101/2023.02.02.525347)
Supplement: Supplement 1 [file media-1.pdf]

Membrane free-energy landscapes derived from atomistic dynamics explain nonuniversal cholesterol-induced stiffening – Supplementary Information

Giacomo Fiorin, Lucy R. Forrest and José D. Faraldo-Gómez

	**Unbiased (single replica)**			**Biased (multiple replicas)**		
**# lipids**	**1800**	**800**	**200**	**1800**	**800**	**200**
**Composition**						
POPC	5.3 μs	5.9 μs	3.3 μs	13 × 0.3 μs	12 × 0.6 μs	12 × 1.5 μs
POPC/CHOL	8.9 μs	9.1 μs	3.4 μs	13 × 0.7 μs	14 × 0.7 μs	13 × 1.5 μs
DOPC	2.0 μs	6.5 μs	3.3 μs	15 × 0.1 μs	14 × 0.7 μs	13 × 1.5 μs
DOPC/CHOL	6.2 μs	7.5 μs	3.4 μs	12 × 0.7 μs	15 × 0.6 μs	13 × 1.5 μs

Table S1: Simulation lengths for phospholipid bilayers at atomistic resolution used in this work. System sizes range from ~50,000 atoms (200-lipid bilayers) to ~660,000 atoms (1800-lipid bilayers). For biased simulations with applied curvature, multiple replicas were simulated using the umbrella-sampling protocol and one replica for each window; the number of windows is reported along with the simulation length of each.

	**MD, bending fluctuations (this work)**			**MD, tilt fluctuations [1]**			**X-ray diffraction [2]**		
	POPC								
k_c (kcal/mol)	17.0	[13.5,	22.1]	19.1	[17.9,	20.3]	11.9	[9.3,	14.5]
k_t (kcal/mol/Å^2)	0.102	[0.084,	0.127]	0.079			0.099	[0.051,	0.147]
	POPC/CHOL								
k_c (kcal/mol)	44.4	[23.7,	99.8]	**P = 0.002** (**)					
k_t (kcal/mol/Å^2)	0.085	[0.072,	0.101]	$P = 0.16$					
	DOPC								
k_c (kcal/mol)	18.0	[13.0,	26.2]	17.1	[15.9,	18.3]	11.6	[10.8,	12.4]
k_t (kcal/mol/Å^2)	0.103	[0.080,	0.138]	0.092			0.128	[0.116,	0.140]
	DOPC/CHOL								
k_c (kcal/mol)	28.5	[16.0,	60.3]	$P = 0.24$					
k_t (kcal/mol/Å^2)	0.081	[0.064,	0.106]	$P = 0.20$					

Table S2: Bending moduli k_c and tilt moduli k_t estimated from membrane bending fluctuations. The estimated mean of each parameter, obtained by fitting Eq. 6 against the data, is reported along with the corresponding 95% confidence interval (CI) in brackets. Bending fluctuation results are from this work (Fig. 7), with uncertainty estimated by parametric bootstrapping [3]. The same distributions were used to perform statistical tests against the hypothesis that cholesterol has no effect, with the resulting P-values reported in the table. Also reported are previous results for the pure lipids using tilt fluctuations [1] and X-ray diffraction [2], with 95% CIs calculated from the reported standard errors, if available.

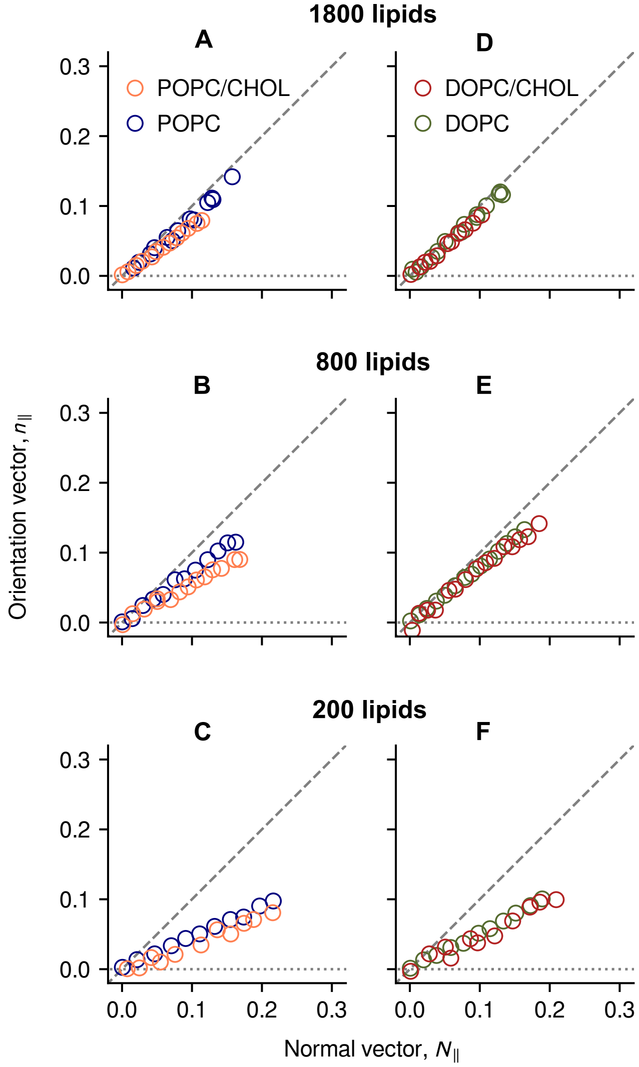

Figure S1: Change in lipid orientation as a function of curvature. The longitudinal component of the mean lipid orientation vector $n_{\|}$ was regressed onto the longitudinal component of the membrane normal vector, $N_{\|}$. Based on this regression, the value of $n_{\|}$ corresponding to the largest value of $N_{\|}$ in each bilayer is plotted for each umbrella-sampling simulation window. Grey dashed lines indicate the Helfrich-Canham theoretical prediction ($n_{\|} = N_{\|}$); dotted lines indicate no changes in lipid orientation ($n_{\|} = 0$). Results are shown for POPC and POPC/CHOL bilayers *(A-C)* and DOPC and DOPC/CHOL bilayers *(D-F)*. The values of the regression slopes are plotted in Fig. 3D,E and Fig. S2.

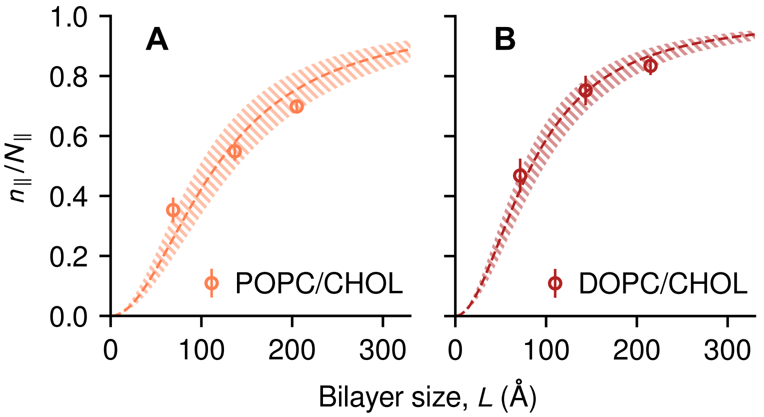

Figure S2: Analysis of the balance between bending and tilt energies for mixed bilayers. The expression in Eq. 3 is fitted against the values of $n_{\parallel}/N_{\parallel}$ for POPC/CHOL *(A)* and DOPC/CHOL bilayers *(B)*, using k_c/k_t as the single fitting parameter. Dashed lines indicate best-fit curves, and striped bands the 95% CI around each curve.

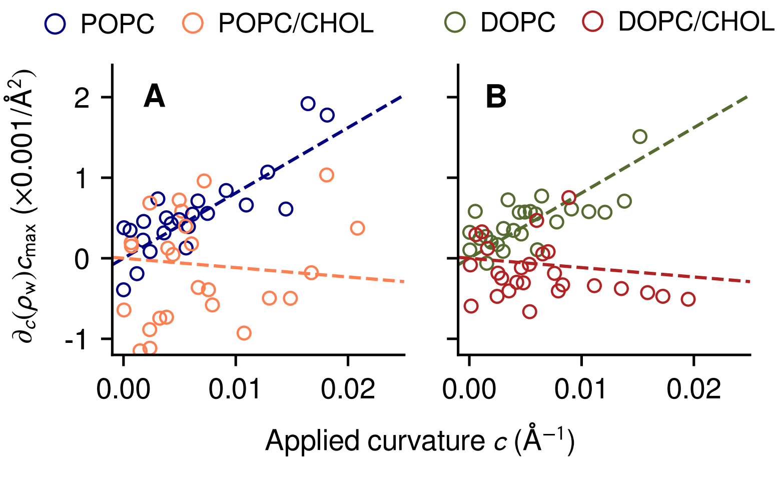

Figure S3: Changes in number of water molecules ρ_w embedded in the hydrophobic section of each bilayer quantified for POPC and POPC/CHOL *(A)*, and DOPC and DOPC/CHOL *(B)*. Dashed lines indicate the results of linear fits of $\Delta\rho_w$ vs. the curvature c, performed on aggregated data from POPC and DOPC (slope = 0.081 ± 0.005 Å^{-2}) or from POPC/CHOL and DOPC/CHOL (slope = -0.011 ± 0.009 Å^{-2}). No significant differences were detected between POPC and DOPC or between POPC/CHOL and POPC/CHOL.

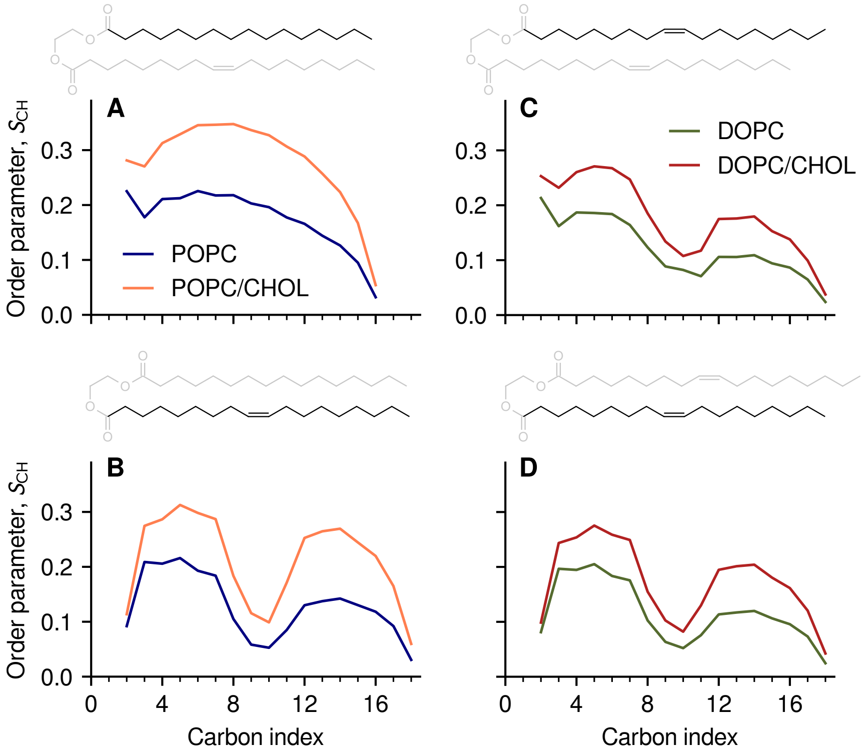

Figure S4: Atomic order parameters S_{CH} between acyl chain carbons and their bound hydrogens, computed as a function of the carbon atom's position along each chain. Results are shown for the *sn*-1 *(A)* and *sn*-2 chain *(B)* of POPC molecules, and for the *sn*-1 and *sn*-2 chains of DOPC molecules *(C-D)*. The chemical structure of each chain is shown alongside the order parameters' values with the same scale.

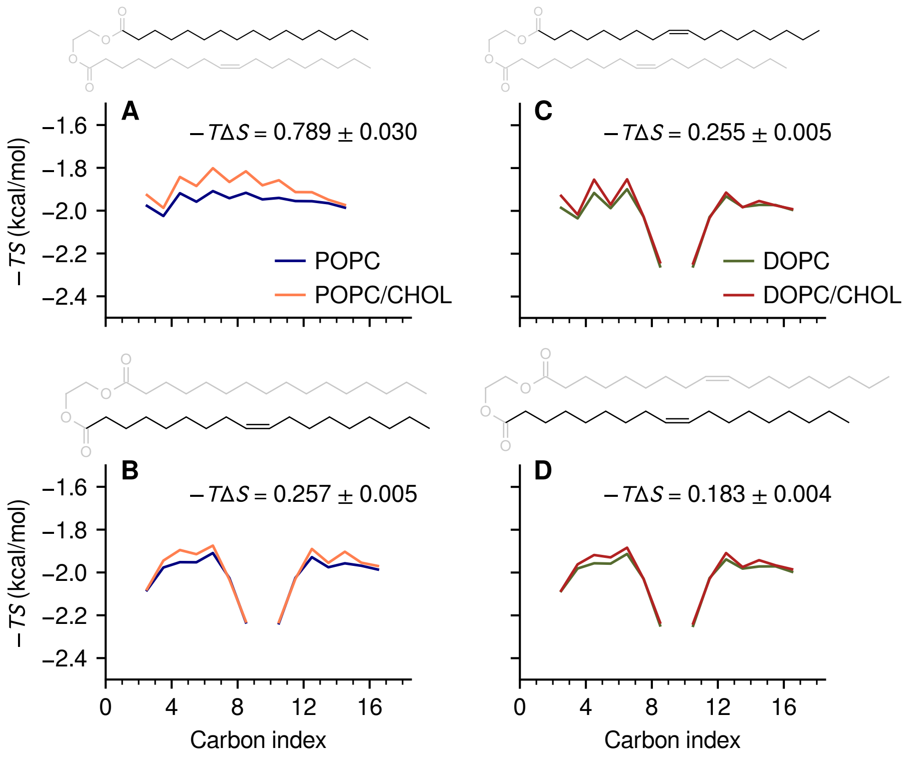

Figure S5: Entropy of lipid acyl chains, extracted from histograms of the torsional angles of carbon atoms with a spacing of 5°. The chemical structure of each acyl chain is shown alongside the reported entropy values as a function of the position along the chain of the second carbon atom of the torsional angle. Entropy values are shown as free-energy contributions, $-TS$, in kcal/mol units. Results are shown for the *sn*-1 *(A)* and *sn*-2 chain *(B)* of POPC molecules, for the *sn*-1 and *sn*-2 chains of DOPC molecules *(C-D)*. For each chain, the aggregated change $\langle -T\Delta S \rangle$ upon adding cholesterol is also reported in the panel (in kcal/mol units).

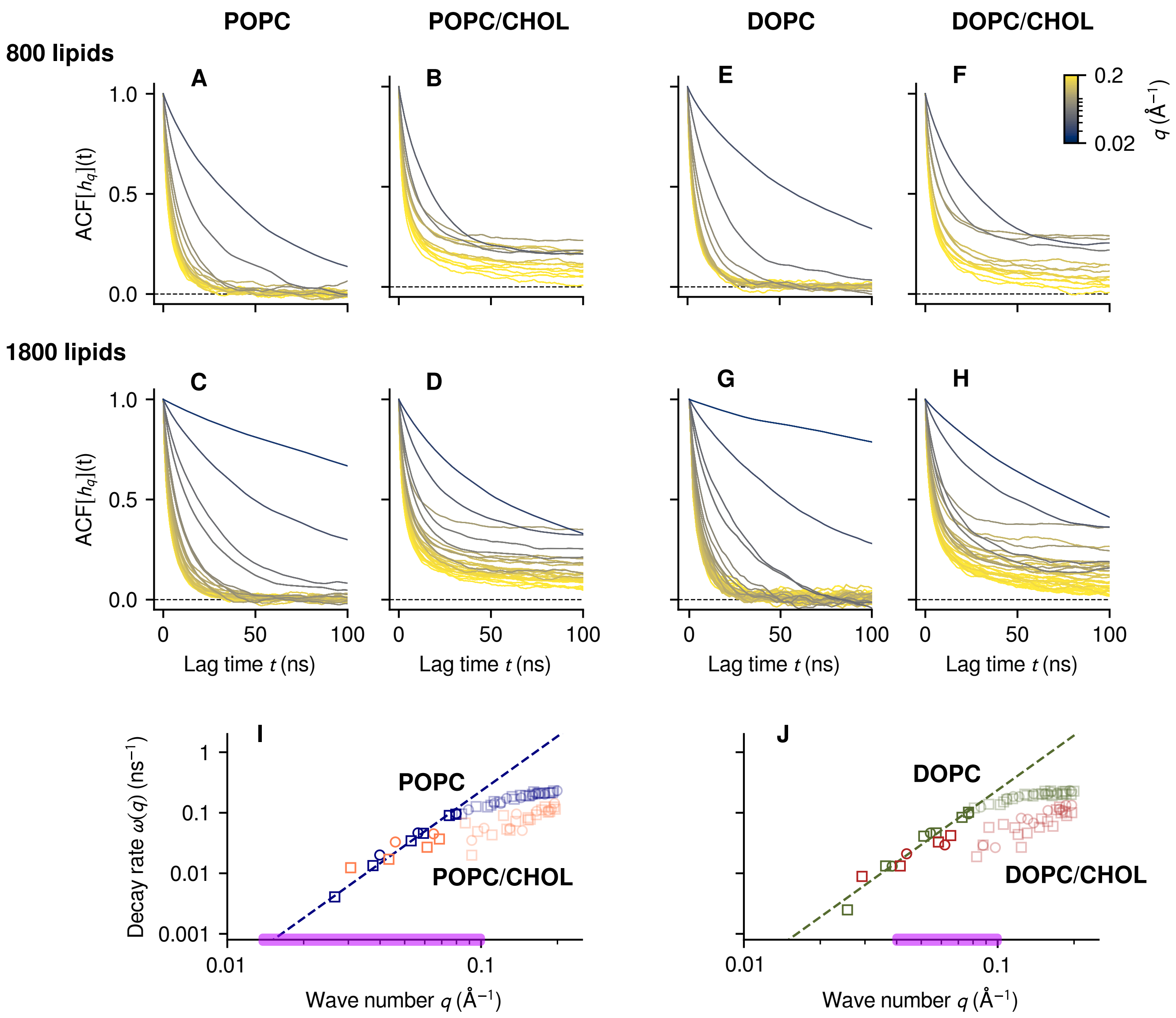

Figure S6: *(A-H)* Auto-correlation functions (ACFs) of the bending Fourier coefficients $h_\mathbf{q}$ for multiple bilayers, colored according to the value of q as in the scale shown. *(I,J)* Decay rates $\omega(q)$, obtained by fitting ACFs to single exponentials, for POPC (blue), POPC/CHOL (orange), DOPC (green) and DOPC/CHOL (red). Decay rates from 800-lipid and 1800-lipid bilayers are shown as circles and squares, respectively; values for $q > 0.8$ Å^{-1} are shown in transparent colors. Dashed lines indicate a linear fit for DOPC and POPC of $\omega(q)$ vs. q^3 as predicted by dynamic theories [4, 5, 6, 7] based on the Helfrich-Canham model [8, 9]. The intervals of q where NSE experiments were carried out [10, 11] are highlighted in purple.

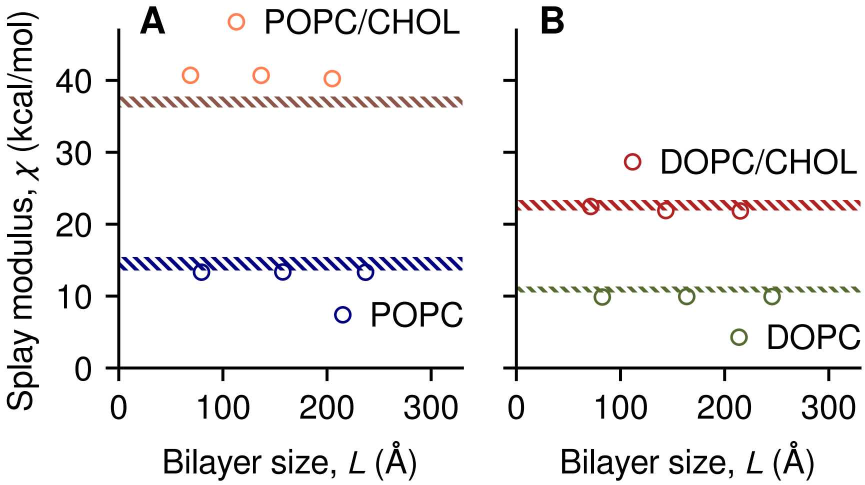

Figure S7: Lipid splay modulus χ computed from fluctuations of the mutual angles $\mathbf{n}_i \cdot \mathbf{n}_j$ between orientation vectors of lipid molecules [12]. *(A)* Values of χ for POPC (blue) and POPC/CHOL (orange) bilayers shown as circles; dashed bands areas indicate the 95% CIs of literature values [12]; because no values for POPC/CHOL were reported, data for the similar mixture POPC/POPS/CHOL 34:30:36 [12] are shown in brown. *(B)* Values of χ for DOPC and DOPC/CHOL, compared to published 95% CIs [11].

References

[1] Venable RM, Brown FLH, Pastor RW, Mechanical properties of lipid bilayers from molecular dynamics simulation. *Chem. Phys. Lipids* **192**, 60–74 (2015).

[2] Nagle JF, Experimentally determined tilt and bending moduli of single-component lipid bilayers. *Chem. Phys. Lipids* **205**, 18–24 (2017).

[3] Ergüder MF, Deserno M, Identifying systematic errors in a power spectral analysis of simulated lipid membranes. *J. Chem. Phys.* **154**, 214103 (2021).

[4] Messager R, Bassereau P, Porte G, Dynamics of the undulation mode in swollen lamellar phases. *J. Phys.* **51**, 1329–1340 (1990).

[5] Seifert U, Langer SA, Viscous modes of fluid bilayer membranes. *Europhys. Lett.* **23**, 71–76 (1993).

[6] Zilman AG, Granek R, Undulations and dynamic structure factor of membranes. *Phys. Rev. Lett.* **77**, 4788–4791 (1996).

[7] Watson MC, Peng Y, Zheng Y, Brown FLH, The intermediate scattering function for lipid bilayer membranes: From nanometers to microns. *J. Chem. Phys.* **135**, 194701 (2011).

[8] Canham P, The minimum energy of bending as a possible explanation of the biconcave shape of the human red blood cell. *J. Theoret. Biol.* **26**, 61–81 (1970).

[9] Helfrich W, Elastic properties of lipid bilayers: theory and possible experiments. *Z. Naturforsch. C* **28**, 693–703 (1973).

[10] Arriaga LR, et al., Stiffening effect of cholesterol on disordered lipid phases: A combined neutron spin echo + dynamic light scattering analysis of the bending elasticity of large unilamellar vesicles. *Biophys. J.* **96**, 3629–3637 (2009).

[11] Chakraborty S, et al., How cholesterol stiffens unsaturated lipid membranes. *Proc. Natl. Acad. Sci. USA* **117**, 21896–21905 (2020).

[12] Doktorova M, Harries D, Khelashvili G, Determination of bending rigidity and tilt modulus of lipid membranes from real-space fluctuation analysis of molecular dynamics simulations. *Phys. Chem. Chem. Phys.* **19**, 16806–16818 (2017).
